# Balanced Essential Amino Acids as Synergistic Therapeutic Agents in Resistance Training: Mechanistic and Clinical Perspectives on Muscle and Metabolic Health

**DOI:** 10.3390/nu18121990

**Published:** 2026-06-19

**Authors:** Jiwoong Jang, Robert R. Wolfe, Il-Young Kim

**Affiliations:** 1 Integrative Metabolic Fluxomics Lab, Lee Gil Ya Cancer and Diabetes Institute, Gachon University, Incheon 21999, Republic of Korea; korea1128@gmail.com; 2 Division of Molecular Medicine, College of Medicine, Gachon University, Incheon 21936, Republic of Korea; 3 Department of Geriatrics, Center for Translational Research in Aging and Longevity, Donald W. Reynolds Institute on Aging, University of Arkansas for Medical Sciences, Little Rock, AR 72205, USA; rrw2@live.com

**Keywords:** essential amino acids, resistance exercise, protein turnover, stable isotope tracer methodology, physical function

## Abstract

Declines of skeletal muscle mass and functions are implicated in the progression of various clinical conditions such as cancers, obesity, insulin resistance, diabetes, and osteoporosis. While no effective and safe drugs against muscle wasting, such as sarcopenia and disease-associated cachexia, have been discovered, it is well documented that dietary essential amino acids (EAAs) or high-quality protein work synergistically to enhance the anabolic effect of resistance exercise training (RT), leading to gains in muscle mass, strength, and muscle quality. Dietary EAAs serve as precursors and signaling molecules for the synthesis of new muscle proteins (both contractile and mitochondrial) and stimulate neuromuscular junction remodeling. Furthermore, EAAs consumed in the post-absorptive state improve endurance capacity via stimulation of mitochondrial biogenesis (independent of PGC1-α) and mitochondrial dynamics (mitochondrial protein synthesis and fission). Here, we discuss (1) traditional molecular mechanisms regulating the muscle proteome through constant turnover (synthesis and breakdown), (2) novel mechanisms by which dietary supplementation of EAAs during RT simultaneously improves muscle strength and endurance, (3) stable isotope tracer methodologies that enable understanding of the dynamic muscle proteome and accurate assessment of functional muscle mass, and finally, (4) clinical implications of combined EAA and RT interventions in the context of muscle and metabolic dysfunction, including sarcopenia, cachexia, obesity, and chronic disease. Collectively, current evidence underscores the potential of balanced EAAs, particularly when combined with resistance training, as a safe, effective, and translationally relevant nutritional strategy to preserve and enhance muscle and metabolic health across healthy and clinical populations.

## 1. Introduction

Skeletal muscle accounts for ~40% of total body mass and ~60% of the total body protein pool [1]. Beyond its primary role in body movement, skeletal muscle is essential for whole-body metabolic homeostasis, functioning as an endocrine organ producing myokines [2], supplying amino acids during catabolic states, and maintaining glucose metabolism. Preservation of adequate skeletal muscle mass and function is therefore of critical importance, as declines in muscle strength and physical function are directly related to morbidity and mortality [3].

Essential amino acids (EAAs) and resistance exercise (RE) are well-known potent anabolic stimuli for regulating muscle mass. RE stimulates muscle protein synthesis (MPS), but the net protein balance (NPB) remains negative because muscle protein breakdown (also stimulated by RE) remains greater than MPS until the provision of suitable nutrition. EAAs or protein (containing high EAAs) alone can induce positive NPB by stimulating MPS without typically affecting muscle protein breakdown (MPB) [4]. Like the beneficial effects on muscle mass, EAAs and RT synergistically improve muscle strength, partly through neuromuscular junction (NMJ) remodeling [5], which may enhance neurotransmission for muscle force generation [6], in addition to muscle hypertrophy through the stimulation of net MPS (i.e., MPS > MPB). Importantly, EAAs serve as anabolic stimuli (mainly through leucine, an EAA), not only for the synthesis of contractile proteins but also for mitochondrial proteins, potentially improving endurance capacity (i.e., cardiorespiratory fitness). In this regard, EAA supplementation in mice improved endurance capacity via stimulation of mitochondrial biogenesis [7].

In this review, we discuss (1) the regulation of muscle mass by RE and EAAs and the underlying key well-known mechanisms, (2) novel potential mechanisms by which dietary supplementation of EAAs during RT induce simultaneous improvements of both muscle strength/quality and endurance capacity, and (3) a stable isotope tracer methodology that enables a better understanding and quantification of muscle protein kinetics in vivo and changes in functional muscle mass. We further address the clinical implications of combined EAA and RT interventions. Unlike previous reviews that have addressed EAAs or RT in isolation, the present review uniquely synthesizes their synergistic mechanistic basis with clinical implications across multiple muscle-wasting conditions and incorporates D_3_-creatine dilution as a more accurate measure of functional muscle mass compared with conventional LBM-based approaches. This structure reflects our view that a mechanistic understanding of EAA-RT interactions is prerequisite for interpreting clinical outcomes and accurately measuring them.

## 2. Methodology

Relevant studies were primarily identified through PubMed and Web of Science searches using combinations of the following keywords: “essential amino acids,” “resistance training,” “muscle protein synthesis,” “neuromuscular junction,” “mitochondrial biogenesis,” “stable isotope tracer,” “sarcopenia,” and “cachexia.” Boolean operators (“AND,” “OR”) were used to refine and expand the search scope. Both human and animal studies published in English up to December 2024 were considered. Preference was given to peer-reviewed original research and review articles examining the effects of essential amino acid supplementation, resistance training, or their combined interventions on muscle metabolism, function, and clinical outcomes. Studies reporting neutral or conflicting findings were also considered to ensure a balanced representation of the available evidence. Additional references were included based on their relevance and conceptual contribution to the topic. Together, this approach ensured a comprehensive yet focused synthesis of current knowledge on the interactive effects of EAAs and resistance training.

## 3. Regulation of Muscle Mass by Physiological Anabolic Stimuli

Dietary EAAs or RE are well-known stimuli for increasing MPS, and EAAs work synergistically with the anabolic effect of RE [8]. In this section, we briefly summarize traditionally well-known key molecular pathways implicated in the regulation of muscle protein turnover and thus muscle mass and quality by EAAs and RE.

### 3.1. Regulation of Muscle Protein Kinetics by EAAs and RE

**Figure 1:**
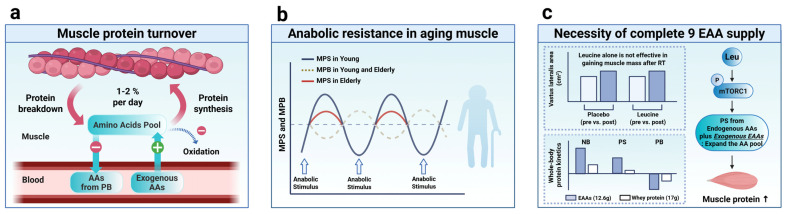
Muscle protein turnover, anabolic resistance, and effects of balanced EAA supplementation. (**a**) Protein turnover involves continuous cycles of Wb-PS and Wb-PB. (**b**) Older adults show a blunted MPS response to RE and EAA intake, termed anabolic resistance. ((**c**), **top**) Leucine alone does not improve hypertrophy during RT [12]. ((**c**), **bottom**) Balanced EAA mixtures enhance NPB more effectively than whey protein by stimulating Wb-PS and suppressing Wb-PB, underscoring the need for all nine EAAs [13]. PB, protein breakdown; Wb-Pb, whole-body protein breakdown; Wb-PS, whole-body protein synthesis; PS, protein synthesis; MPS, muscle protein synthesis; MPB, muscle protein breakdown; RT, resistance exercise training; NB, net balance; Leu, leucine; AA, amino acid; EAA, essential amino acid; mTORC1, mammalian target of rapamycin complex 1. In (**a**), the green (+) symbol indicates amino acids entering the intracellular pool (from exogenous intake), and the red (−) symbols indicate amino acid efflux from the pool (via oxidation) and the catabolic origin of amino acids derived from protein breakdown (PB). Created in BioRender. Jang, J. (2026) https://BioRender.com/uf0iwdb (accessed on 16 June 2026).

Muscle protein turnover and anabolic stimuli: In the postabsorptive state, MPB usually exceeds MPS, resulting in a negative net protein balance (NPB) over time if there is no intervention [9]. RE and EAA intake elevate MPS, but RE alone does not yield positive NPB unless there is an adequate availability of EAAs from the consumption of high-quality protein or EAAs, which counters the persistent elevated rate of MPB after exercise [10]. Protein or EAA ingestion alone can transiently stimulate MPS and shift the balance toward net synthesis, even in the absence of exercise. This response is primarily driven by EAAs, since non-essential amino acids (NEAAs) do not enhance MPS when added to EAA mixtures (Figure 1a) [11].

Anabolic resistance in aging muscle: In older adults, the muscle’s ability to respond to anabolic stimuli such as RE and EAA intake becomes diminished. This phenomenon, known as anabolic resistance, results in a lower MPS response. Although basal protein turnover rates in the fasted state are similar between younger and older adults, the anabolic response to a modest EAA intake is delayed and attenuated in older individuals [14]. This resistance can be overcome with higher doses of protein or EAAs [15]. Similarly, MPS following RE is also lower in older adults compared to younger individuals, even when exercise volume is controlled [16]. This may be partly due to reduced activation of the mTORC1 pathway in older adults in response to anabolic stimuli, which plays a central role in initiating protein synthesis (PS) [17]. Studies have shown that leucine-enriched EAA formulations can enhance MPS and help overcome the age-related resistance, both at rest and post-exercise (Figure 1b) [8].

Necessity of complete 9 EAA supply: While leucine can trigger mTORC1 signaling, complete PS requires all EAAs. Without adequate availability of each EAA, the whole-body protein synthesis (Wb-PS) process cannot proceed efficiently. Clinical trials showed that leucine supplementation alone did not improve lean body mass (LBM) or strength in normal [12] and in clinical conditions [18]. On the other hand, balanced EAAs have been more effective in promoting muscle mass, with or without exercise interventions [19]. Additionally, balanced EAA mixtures often result in greater whole-body anabolic responses than intact protein sources, likely due to their optimized composition and faster absorption (Figure 1c) [20].

### 3.2. Molecular Signaling Pathways Implicated in MPS and MPB Regulation

#### 3.2.1. Muscle Protein Synthesis

**Figure 2:**
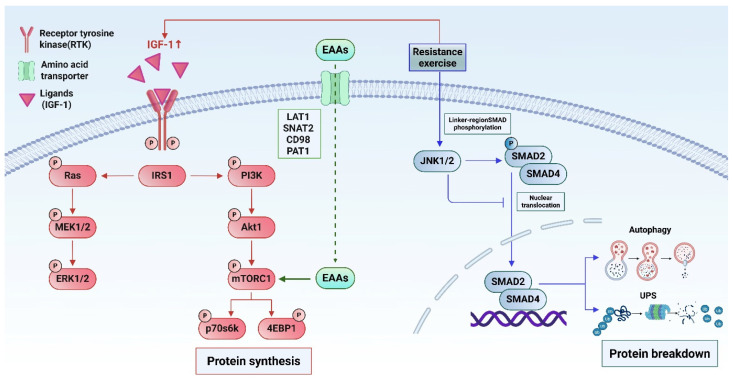
Major signaling pathways implicated in the stimulation of muscle protein synthesis in response to resistance exercise or EAA supplementation. MAPK/ERK1/2 pathway: IGF-1 activates IGF-1R, leading to IRS1–Ras–MEK1/2 signaling, ERK1/2 activation, and protein synthesis. mTORC1–p70S6K pathway: IGF-1 binding activates IRS1–PI3K–Akt1, which stimulates mTORC1 to phosphorylate p70S6K and 4E-BP1. Amino acid transporters: RE or EAA supplementation increases expression of LAT1, SNAT2, CD98, and PAT1, enhancing intracellular amino acid availability to activate mTORC1 and support synthesis. JNK/SMAD pathway: Mechanical stress induces JNK1/2-mediated phosphorylation of SMAD2, preventing SMAD2/4 nuclear translocation and suppressing autophagy and UPS-mediated breakdown. Abbreviations: RTKs, receptor tyrosine kinases; IRS1, insulin receptor substrate 1; PI3K, phosphoinositide 3-kinase; Akt1, protein kinase B; mTORC1, mammalian target of rapamycin complex 1; p70S6K, ribosomal S6 kinase 1; 4E-BP1, eukaryotic translation initiation factor 4E-binding protein 1; MEK1/2, MAPK kinase 1/2; ERK1/2, extracellular signal-regulated kinase 1/2; JNK1/2, c-Jun N-terminal kinase 1/2; SMAD2/4, SMAD family members 2 and 4; UPS, ubiquitin–proteasome system; LAT1, L-type amino acid transporter 1; SNAT2, sodium-dependent neutral amino acid transporter 2; CD98, cell-surface glycoprotein; PAT1, proton-coupled amino acid transporter 1. Red arrows indicate activation or signal propagation; the green dashed arrow indicates amino acid transport and the contribution of EAAs to mTORC1; the blunt-ended line (⊥) denotes inhibition; encircled P denotes phosphorylation. Created in BioRender. Jang, J. (2026) https://BioRender.com/p4hwc3j (accessed on 16 June 2026). Key references supporting the pathways depicted: mTORC1 pathway [21,22,23]; ERK1/2 pathway [24,25]; JNK/SMAD pathway [26].

MPS is regulated by a complex but highly coordinated network of intracellular signaling pathways integrating mechanical, hormonal, and nutritional cues. Among these, (1) mTORC1, (2) ERK1/2, and (3) JNK/SMAD signaling pathways interact to optimize MPS, particularly in response to RE and EAAs (Figure 2).

The mTORC1 pathway: The mTORC1 pathway serves as a central hub for PS. Activated downstream of Akt1, mTORC1 stimulates MPS through phosphorylation of p70s6k1 and 4E-BP1. Both RE and EAAs (particularly leucine enriched) robustly activate mTORC1 signaling and enhance MPS, with combined stimulation yielding greater effects than alone [21]. Furthermore, mTORC1 activation upregulates amino acid transporters (LAT1, SNAT2, CD98, PAT1), increasing intracellular AA availability and reinforcing anabolic signaling by EAAs [22] and RE [23]. This upregulation enhances AA availability, leading to the stimulation of MPS not only by providing building blocks but also by activating mTORC1 signaling for the synthesis of new proteins [22].

The ERK1/2 pathway: The ERK1/2 pathway, upon activation as a downstream of receptor tyrosine kinases (RTKs) such as those triggered by IGF-1, initiates a classical MAPK cascade involving Ras, Raf, and MEK1/2. Once activated, ERK1/2 enhances protein translational capacity by stimulating ribosomal RNA gene expression [24] and phosphorylating protein translation initiation factors such as eIF4E [27], and has been shown to respond to RE in humans [14] and EAAs in cell lines [25]. Moreover, ERK1/2 may function in parallel with or upstream of mTORC1, as both pathways converge on components of the translational machinery, suggesting potential crosstalk that amplifies anabolic signaling.

The JNK/SMAD pathway: The JNK/SMAD pathway represents another axis through which MPS is indirectly regulated. Myostatin, a key negative regulator of muscle mass, signals through SMAD2/3 to promote muscle atrophy [28]. RE activates JNK, which inhibits this catabolic signal by phosphorylating the SMAD2 linker region, thereby preventing its nuclear translocation and transcriptional activity [26]. Importantly, inhibition of SMAD signaling is associated with enhanced mTORC1 activity [29], indicating that JNK-mediated suppression of myostatin may reinforce mTORC1-driven PS. Though the role of EAAs in modulating JNK/SMAD signaling remains underexplored, the downstream effect of myostatin inhibition clearly intersects with nutrient-sensitive anabolic pathways.

Taken together, these respective pathways, although mechanistically distinct, converge to enhance MPS: ERK1/2 increases translational capacity, JNK/SMAD reduces inhibitory input, and mTORC1 integrates and amplifies both mechanical and nutrient signals. Their interaction provides a robust molecular framework for skeletal muscle anabolism in response to RE and EAA intake.

#### 3.2.2. Muscle Protein Breakdown

Skeletal muscle mass is determined not only by MPS but also by the balance between MPS and MPB. MPB is essential for removing damaged or dysfunctional proteins, making it a critical aspect of muscle proteostasis. In catabolic states such as severe burns, both MPB and MPS are elevated. Increased MPB raises intracellular amino acid availability, which in turn activates MPS but is insufficient to prevent muscle loss [28,30]. This reflects the close interplay between the two protein kinetic processes. The maintenance of muscle health requires a constant state of MPS and MPB in an orchestrated manner. MPB is primarily regulated by the (1) ubiquitin–proteasome system (UPS) and the (2) autophagy–lysosome system.

The ubiquitin–proteosome system: The UPS selectively degrades proteins via E1–E2–E3 enzymes. In muscle, Atrogin-1 and MuRF1 are key E3 ligases targeting proteins involved in translation and structural integrity [28]. Acute RE increases their expression transiently [31], while RT and EAAs reduce them [32]. EAAs, particularly with myostatin inhibition, lower Atrogin-1 mRNA and ubiquitinated protein levels even under dexamethasone (DEX)-induced atrophy [33,34].

The autophagy–lysosome system: Along with the UPS, the autophagy–lysosome system contributes to muscle proteostasis by mediating MPB. Traditionally viewed as a non-selective degradation pathway for bulk and long-lived proteins, autophagy is now known to include selective forms, such as mitophagy, which targets damaged mitochondria [35]. RE activates autophagy [36], likely to support muscle remodeling and metabolic health. EAAs suppress autophagy markers like LC3-II/LC3-I and ATG7 in atrophic conditions, but RE offsets this suppression, supporting balanced protein turnover [34].

In summary, MPB is a regulated process that maintains muscle quality and function. Coordinated modulation of UPS and autophagy by RE and EAAs ensures dynamic proteostasis and supports muscle adaptation.

#### 3.2.3. Suitable Rates of Protein Turnover Are Key to the Maintenance of Muscle Quality

Muscle function depends on both muscle mass and muscle quality, which is defined as functional capacity normalized to muscle mass. Protein turnover, involving MPS and MPB, maintains muscle quality by replacing damaged proteins and synthesizing new ones. Disruption in this balance leads to rapid declines in muscle quality. In a 28-day bed rest study, reduced protein turnover was associated with losses in muscle strength and power, particularly when cortisol levels were increased by infusion, even before the loss of LBM [37]. EAA supplementation during bed rest preserved function, especially in soleus type I and vastus lateralis type II fibers. The response to EAAs during bed rest highlights the role of nutritional interventions in maintaining muscle quality. Our lab also showed that EAAs and RT affect MPB differently depending on the physiological context [34]. Under anabolic conditions, EAAs lowered Atrogin-1 without changing MuRF1 or autophagy activity, indicating selective inhibition of proteolysis. Under catabolic stress, such as DEX treatment, EAAs broadly reduced protein degradation markers, including LC3II/I and ATG7. Intracellular EAA levels regulate turnover and are supplied through diet or MPB. These findings support the concept that maintaining balanced protein turnover through RE and EAA supplementation is critical for preserving muscle quality in both healthy and catabolic conditions.

## 4. New Insight into the Role of Dietary Essential Amino Acids: Amplification of Resistance Training Adaptation and Beyond

RT is generally known to have little or even negative impact on endurance capacity. While providing distinct health benefits, concurrent exercise training of both modes of exercise, i.e., RT with endurance exercise training (ET), compromise the training adaptation specific to each mode (i.e., ET for endurance and RT for muscle hypertrophy), which is termed the “interference effect” [38]. However, owing to their dual role as anabolic stimulus via mTORC1 activation and as a precursor for MPS, free EAAs can bypass such interference without hindering adaptations to either RT or ET. They also stimulate mitochondrial biogenesis by promoting the synthesis of mitochondrial proteins, thereby enhancing endurance capacity alongside muscle strength and mass [5]. Therefore, combining RT with a free EAA supplement (containing all EAAs in optimal ratios) may represent an effective strategy to simultaneously improve muscle strength, endurance, and metabolic health. In the following section, we discuss the potential mechanisms through which free EAAs and RT synergistically improve physical performance (both strength and endurance) and metabolic function (e.g., insulin sensitivity).

### 4.1. Muscle Strength and Quality

Muscle strength and cardiorespiratory fitness (i.e., endurance capacity) are the two best indicators for the risk of all-cause morbidity and mortality [39]. Therefore, efforts to improve or at least maintain muscle strength as well as endurance are of critical importance not only for athletic performance but also for general and clinical conditions. For this purpose, RT is known to be the most powerful natural means that can improve muscle strength [38]. In addition, EAAs are another effective means for this purpose, as EAAs increase not only the efficacy of RT on gains in muscle mass and strength but also for enhancing muscle quality (i.e., muscle strength/muscle mass), especially when combined with RT [5,33]. Changes in muscle quality often precede changes in muscle mass, as evidenced by sarcopenic older adults [40] and those performing RT [41]. Although the mechanisms remain unclear, alterations in muscle protein turnover and NMJ stability may play important roles in regulating both muscle strength and quality.

#### 4.1.1. Muscle Protein Turnover

Age-associated muscle loss (i.e., sarcopenia) alone does not account for the observed declines in muscle strength and physical function, as muscle strength decreases faster than that of muscle mass, although this conclusion is based on LBM [42]. Reduced muscle quality (muscle strength/mass) with aging seems to be due in part to the age-associated decline in muscle protein turnover, driven by physical inactivity, comorbidity, and/or aging [43]. This age-associated decline in muscle protein turnover may result in the accumulation of non-functional, low-quality proteins in muscles, potentially leading to decreases in quality in aging muscle. Supporting this notion, dietary EAAs improved muscle strength and physical function in older adults without significant changes in LBM [44], suggesting a role for enhanced muscle protein turnover in improving muscle quality. Furthermore, EAA supplementation reversed strength loss after 10 days of bed rest despite a persistent loss of LBM [45]. Since contractile protein occupies a large proportion of the skeletal muscle protein pool (~90%), an increase in Wb-PS of contractile proteins can have a significant effect on muscle mass and strength. Indeed, an increase in synthesis of contractile proteins highly correlated with muscle strength, highlighting the potential role of EAAs [46] to improve muscle quality by increasing the contractile protein turnover.

#### 4.1.2. Neuromuscular Junction Stability

Previously, we reported that structural improvement in the neuromuscular junction (NMJ) is related to gains in muscle strength, independent of changes in muscle mass (i.e., improved muscle quality) [33]. It is possible that structural improvements in NMJ, as indicated by increased acetylcholine receptor (AchR) clustering, can lead to an increase in muscle strength in part through enhanced neural transmission in the motor unit (alpha-motor neuron and the muscle fibers innervated). Supporting this, Chevessier et al. reported that genetic mutations in muscle-related receptor tyrosine kinase (Musk), a marker of NMJ structural stability, reduce muscle strength and muscle quality [47]. In addition, it has been reported that a pharmacologically enhanced AchR clustering attenuated muscle weakness in congenital myasthenic syndrome (CMS) [6]. Thus, increased muscle quality by dietary EAAs and/or RT may be due in part to an improved NMJ structure and acetyl-choline clustering via activation of the Agrin/MuSK pathway. It should be noted, however, that the evidence supporting this mechanism is primarily derived from preclinical animal models. Consistent with this observation, we recently demonstrated that dietary EAAs reversed DEX-induced decreases in NMJ stability and muscle strength [34]. In addition, combined treatment of EAAs and RT enhanced muscle strength and quality through improved NMJ stability via MuSK activation, even under normal conditions [5]. Whether these findings translate to humans remains to be established, and further human studies are warranted. Together, these results suggest the potential role of dietary EAAs and/or RT in promoting structural improvements in NMJ, thereby enhancing muscle strength and quality. Nevertheless, the specific molecular mechanisms by which EAAs influence the Agrin/MuSK signaling pathway remain to be elucidated, and further mechanistic studies are warranted to clarify these processes.

### 4.2. Endurance Capacity

It is appreciated that different exercise modes produce distinct adaptations (i.e., ET for endurance and RT for muscle hypertrophy and strength) and can counteract each other’s effect [38]. It is, however, of critical importance that both traits (i.e., endurance and muscle strength) be improved or at least maintained well for one’s healthy life. In this regard, the addition of dietary EAAs to RT may be an effective strategy to synergistically achieve these goals as EAAs not only amplify the anabolic potential of RT but also increase rates of synthesis of mitochondrial proteins as well as contractile proteins by serving as precursors for MPS during RT. Here, we will discuss the potential mechanisms by which EAAs improve endurance during muscle-hypertrophying RT, including stimulation of mitochondrial biogenesis and dynamics.

#### 4.2.1. Mitochondrial Biogenesis

Mitochondrial biogenesis refers to the process by which new mitochondrial proteins are synthesized, for which PGC1-α has been considered a primary regulator. However, recent studies have also reported that mitochondrial biogenesis can occur in a PGC1-α-independent manner. For example, muscle-specific PGC1-α KO did not prevent ET-induced mitochondrial biogenesis [48]. Limited studies have explored the effects of EAAs or protein intake on endurance capacity. Robinson et al. reported that 6 weeks of ET with carbohydrate or isocaloric protein beverage after each bout of endurance exercise, VO_2max_, a well-known index for endurance capacity, increased only in the protein-supplemented participants [49]. Similarly, we found that EAA supplementation is effective in improving endurance when taken alone or in combination with RT [5]. Furthermore, EAA supplementation for 2 weeks partially prevented DEX-induced declines in endurance capacity, muscle mass, and muscle strength [34]. Additionally, free EAA consumption (in drinking water) for 4 weeks improved endurance capacity via increased mitochondrial biogenesis in both sedentary and endurance-trained groups [7]. These findings should be interpreted with caution, as direct human evidence linking EAA supplementation to mitochondrial biogenesis-mediated improvements in endurance capacity remains limited. Therefore, identifying and understanding the mechanism(s) through which free EAAs, alone or combination with RT, enhance endurance capacity via mitochondrial biogenesis may be a valuable direction for future research.

#### 4.2.2. Mitochondrial Dynamics

Mitochondrial dynamics, represented by fission and fusion, are essential for maintaining mitochondrial abundance, morphology, and quality. Mitochondrial fission promotes the removal of damaged components, preserving function [50]. With respect to mitochondrial fission, deficiency of the fission-promoting factor dynamin-related protein 1 (DRP1) leads to loss of mitochondrial function and muscle weakness [51]. Conversely, increased DRP1 expression induced via DRP1 plasmid transfection in aged muscle cells increased ATP production and decreased ROS production [52]. However, excessive DRP1 expression in muscle-specific transgenic mice impaired oxidative capacity and endurance [53], indicating that a suitable fission level is essential for mitochondrial and muscle health. Although limited, some evidence links EAAs or RE to DRP1 activation via mTORC1 activation. For example, electric stimulation-induced RE increased DRP1 phosphorylation for up to 1 h [54], and pharmacological mTORC1 inhibition decreased phosphorylated DRP1 (S616) levels without affecting total DRP1 expression [55]. In addition, our recent study demonstrated that free EAA supplementation improved the endurance capacity and mitochondrial function by enhancing the mitochondrial PS rate and mTORC1-DRP1 signaling, with greater effects when combined with RT [5]. These findings are promising; however, as this evidence is derived from a preclinical model, whether EAA and RT regulate mitochondrial dynamics via mTORC1-DRP1 signaling in humans requires further investigation. Therefore, co-treatment of EAA supplementation and RT may better improve mitochondrial quality through enhancement of the mitochondrial protein turnover rate via mTORC1-DRP1 signaling. However, further mechanistic studies are needed to elucidate how EAA and RT regulate mitochondrial dynamics and quality at the molecular and metabolic levels.

### 4.3. Metabolic Health: Regulation of Insulin Sensitivity

Skeletal muscle accounts for ~40–50% of body weight and most (~80%) of postprandial glucose disposal. Thus, preservation of a suitable muscle mass and quality is of critical importance for the maintenance of glucose metabolism. In this regard, RT and EAAs may represent a promising combination to improve insulin sensitivity via gains in muscle mass and quality, though direct clinical evidence in humans remains limited, with most supporting data derived from preclinical models. In our recent study using a DEX-induced muscle wasting mice model, we confirmed that EAAs and/or RT prevented reductions in both muscle strength and endurance capacity and reversed the deterioration of glucose tolerance and insulin sensitivity, with the greatest effect observed in the combined treatment of EAA and RT. We also verified that muscle glucose oxidation was increased by EAAs, RT, or both, as evidenced by increased ^13^C labeling in citrate, the first TCA (tricarboxylic acid) cycle intermediate, derived from ^13^C labeled glucose, compared to DEX alone [34]. Similarly, co-treatment of EAA and RT in mice under normal conditions increased glucose uptake into muscle and systemic glucose metabolism through additional muscle mass gain [5]. These results suggest that the improved physical functions with the combined treatment of EAAs and RT may lead to the improvement of glucose metabolism, or vice versa.

**Figure 3:**
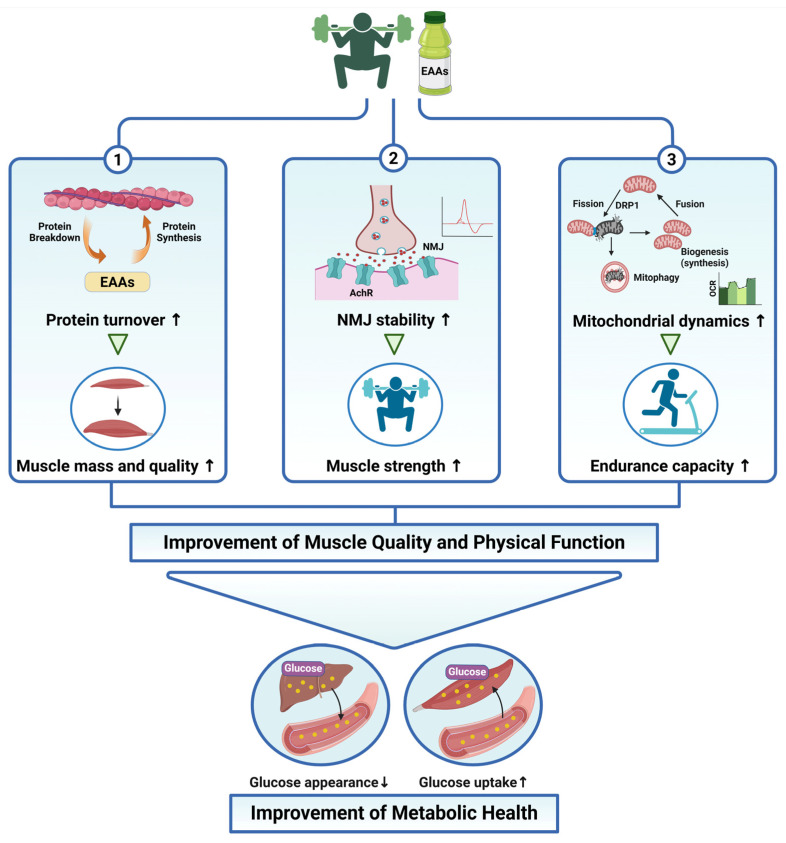
Synergistical effect of EAAs and resistance exercise training for muscle strength/quality, endurance, and metabolic health. The integrated physiological responses include (1) enhanced protein turnover through increased muscle protein synthesis, contributing to improved muscle mass and quality; (2) improved neuromuscular junction (NMJ) stability, supporting greater muscle strength; and (3) upregulated mitochondrial dynamics, encompassing fission, fusion, and biogenesis, thereby increasing endurance capacity. Collectively, these adaptations enhance overall muscle quality and physical function, leading to improved systemic glucose handling, characterized by reduced glucose appearance and increased glucose uptake. The schematic illustrates that the benefits of combined RE and EAA supplementation encompass both functional gains and improvements in metabolic health, beyond muscle hypertrophy. NMJ, Neuromuscular junction; AchR, Acetylcholine receptor; DRP1, Dynamin related protein 1; OCR, Oxygen consumption rate. Upward and downward arrows (↑, ↓) indicate an increase and a decrease, respectively; open (hollow) arrows denote the functional outcome resulting from each mechanism; and the curved double-headed arrow in panel (1) represents the continuous cycle of muscle protein synthesis and breakdown. Created in BioRender. Jang, J. (2026) https://BioRender.com/3tedrjd (accessed on 16 June 2026). Key references supporting the mechanisms depicted: NMJ remodeling [5,33]; mitochondrial dynamics [5,34]; glucose metabolism [5,34].

Taken together, adding dietary free EAAs to RT may induce a simultaneous improvement of both muscle strength/quality and endurance in part through (1) the stimulation of functionally active new proteins, (2) the structural remodeling of NMJ, and (3) an increase in mitochondrial quality, leading to or resulting from enhancements of glucose metabolism and insulin sensitivity (Figure 3).

Table 1 summarizes the principal mechanistic pathways discussed in Section 3 and Section 4 and their corresponding molecular effects, functional outcomes, and clinical relevance.

## 5. Understanding Dynamic Muscle Proteome: Dissection with Stable Isotope Tracers

Body protein is maintained through dynamic proteostasis, where MPS and MPB are tightly regulated under normal conditions. Disruption of this balance can lead to pathological states. In disease-associated cachexia, for example, prolonged elevation of MPB relative to MPS results in severe muscle wasting. Interestingly, the increased MPB also elevates AA availability, which can secondarily stimulate MPS, though insufficient to restore balance [56]. This highlights the importance of assessing both aspects of the dynamic aspects of protein metabolism (i.e., synthesis and breakdown) in vivo. Stable isotope tracer methods using ^13^C, ^15^N, or ^2^H tracers represent the gold standard for evaluating these dynamics under both normal and disease conditions [57,58]. In this section, we briefly describe tracer methodologies used to assess muscle protein turnover and to quantify functional muscle mass, both essential for understanding muscle health.

### 5.1. Body Proteins Are in a Constant State of Turnover: Motion Pictures Differ from Snapshots

An appropriate rate of muscle protein turnover is essential not only for maintaining muscle mass but also for preserving muscle quality. Despite the dynamic nature of the muscle proteome, many researchers rely heavily on snapshot measures such as transcriptomics, proteomics, or phosphorylation levels to infer dynamic processes, often leading to misinterpretation of data from both in humans [59] and animals [60]. Therefore, in the following section, we will discuss a tracer methodology that enables in vivo quantifications of muscle protein kinetics. For more in-depth information, readers are referred to more detailed reviews [57].

### 5.2. Stable Isotope Tracer Methodology Exploring Dynamic Nature of Muscle Proteome

**Figure 4:**
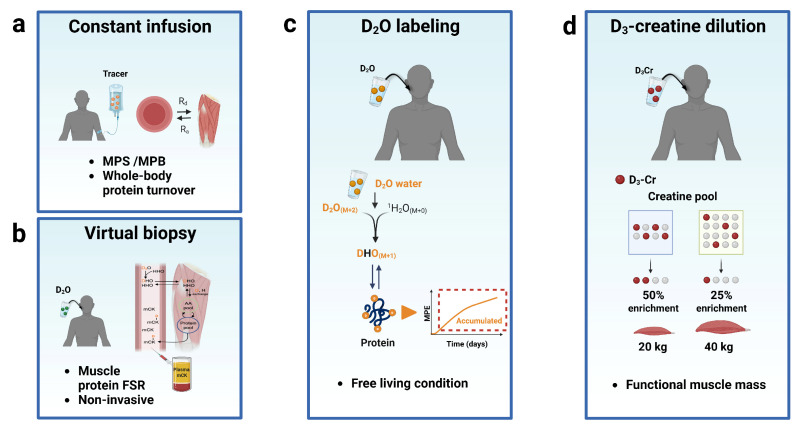
Schematic diagram of assessing muscle protein kinetics using stable isotope tracer methodology. (**a**) Constant infusion of stable isotope-labeled amino acids enables quantification of MPS, MPB, and whole-body protein turnover through kinetic modeling of tracer *R_a_* and *R_d_*. (**b**) Virtual biopsy leverages D_2_O labeling of muscle-enriched proteins, such as mCK, sampled from plasma or urine, offering a non-invasive means to estimate FSR. (**c**) Oral ingestion of D_2_O permits in vivo labeling of newly synthesized proteins over extended time periods under free living conditions. This approach captures accumulated muscle protein synthesis at both the individual and whole-body levels. (**d**) D_3_-creatine dilution, following ingestion of labeled creatine, provides a non-invasive estimate of total functional muscle mass based on the dilution of D_3_-creatinine in urine. Integrating (**c**) and (**d**) allows for a linkage between tracer-derived protein synthesis rates and structural outcomes, facilitating a comprehensive assessment of muscle remodeling and plasticity over time. AA, amino acid; FSR, fractional synthesis rate; *R_a_*, rate of appearance; *R_d_*, rate of disappearance; D_2_O, deuterium oxide; mCK, creatine kinase muscle form; Cr, creatine. Created in BioRender. Jang, J. (2026) https://BioRender.com/3tedrjd (accessed on 16 June 2026). Key references: constant infusion methodology [57,58]; virtual biopsy [62]; D_3_-creatine dilution [64].

Information on in vivo protein dynamics is typically assessed with a primed constant infusion of AA tracer (most commonly using labeled leucine or phenylalanine) [58]. Recently, use of deuterated, or heavy, water labeling methods is increasing due largely to several advantages, including (1) measurement of the true precursor enrichment and (2) assessment of MPS in free living conditions [61]. Traditional MPS assessment requires muscle biopsies, usually from specific muscles like the vastus lateralis. However, newer approaches now allow for whole-body MPS measurement without biopsy, a method referred to as “virtual biopsy” [62]. Moreover, combining heavy water labeling with proteomic technologies enables a detailed assessment of proteome kinetics in vivo [63]. Stable isotope tracers also allow for direct measurement of functional muscle mass using the D_3_-creatine dilution technique [64]. In the following section, we briefly present stable isotope tracer methodologies commonly used to evaluate dynamic muscle proteome turnover and functional muscle mass. These tools are essential for advancing muscle research in both healthy and clinical populations (Figure 4).

#### 5.2.1. Constant Infusion of AA Tracers

While also applicable to other polymers (e.g., fatty acids and DNA) [58], MPS and MPB can be quantified as changes in product labeling with administration of individual labeled AA tracers (such as ^2^H_5_-phenylalanine) and are typically expressed as the fractional synthesis rate (FSR, %/t) and fractional breakdown rate (FBR, %/t). Based on the precursor–product principle, FSR is assessed by measuring the incorporation rate of the AA tracer to protein in muscle [57], and FBR by measuring the dilution of AA tracer enrichment of the protein [65]. Since FSR and FBR are relative measures, they must be multiplied by the total protein pool size to derive absolute MPS and MPB values [58]. Since muscle protein dynamics influence whole-body protein turnover, and vice versa, assessment of both aspects offers important physiological context and helps interpret muscle-specific changes in coordination with systemic metabolic regulation. The constant infusion approach enables the measurement of rapid changes in muscle protein kinetics (over a few hours). Complementary to amino acid tracer infusion techniques, the arterio–venous (AV) balance approach provides simultaneous information on amino acid uptake, release, and transmembrane transport, thereby enabling the assessment of both synthesis and breakdown. Although often applied to evaluate whole-body metabolic flux, this technique can also be used specifically for skeletal muscle, offering unique insights into localized protein dynamics [58,66].

#### 5.2.2. Heavy Water Labeling

The heavy water labeling method is increasingly utilized for assessment of muscle protein FSR over days to weeks to months, with several distinct methodological strengths, including (1) avoiding the true precursor enrichment issue, (2) assessing MPS in free living conditions [61], and (3) simultaneously assessing other polymers, such as DNA, glucose, and triacylglycerol [67]. One major advantage of heavy water labeling is that deuterium from body water is incorporated into NEAAs through normal metabolism, leading to uniform labeling of the intracellular precursor pool directly used for PS. This feature avoids the traditional precursor problem in amino acid tracer infusion studies, where discrepancies arise from using plasma versus aminoacyl-tRNA enrichment as surrogate measures [58]. To measure muscle protein FSR, a small daily dose of heavy water (typically <100 mL) is consumed after priming. The deuterium equilibrates with body water, exchanges with free amino acids via metabolic reactions, and is incorporated into newly synthesized proteins [68]. FSR is then calculated as the rate of labeled precursor incorporation divided by precursor enrichment over time, as described in the tracer incorporation model. Rapid changes in muscle protein FSR are difficult to measure with this method.

#### 5.2.3. Virtual Biopsy: Heavy Water Labeling and Blood Sampling

Assessment of muscle protein FSR typically requires muscle tissue obtained via needle biopsy, a procedure that, while effective, is invasive and difficult to perform in many clinical situations. The “virtual biopsy” technique has been developed to avoid the necessity of obtaining tissue samples to measure MPS. The virtual biopsy method consists of (1) deuterium labeling of muscle proteins and (2) measurement of the label in proteins that are exclusively muscle-derived and released into the bloodstream or urine, such as muscle isoform of creatine kinase (>96% from muscle tissue) [69]. The calculation is identical to that when muscle biopsies are used, except direct determination of the precursor enrichment of the infused AA tracer. This method enables accurate assessment of muscle FSR in both human subjects and animal models [69].

#### 5.2.4. Assessment of Functional Muscle Mass: D_3_-Creatine Dilution Method

Despite the fact that accurate measurement of muscle mass is essential for evaluating the effects of interventions such as EAAs, RT, or pharmacological treatments, most previous studies reported LBM (by DXA, impedance, etc.) or at most muscle volume (by MRI) [70,71], both of which are not muscle mass. Muscle mass constitutes a major portion of LBM that varies widely, from 31–68% in healthy individuals to 8–49% in those with muscle-wasting conditions [72]. Traditional methods are also affected by hydration status and other physiological factors, further limiting their accuracy [73]. The D_3_-creatine dilution method addresses these issues by directly assessing functional muscle mass. Following oral ingestion of a small dose (<70 mg), D_3_-creatine is transported into skeletal muscle, where 98% of body creatine resides. In skeletal muscles, creatine (both labeled and unlabeled) is irreversibly converted to creatinine and then excreted in urine while preserving the isotopic enrichment, defined as the ratio of labeled to total creatinine. This enrichment provides information for estimating total muscle creatine pool size and thus a reliable whole-body functional muscle mass [74]. Therefore, future studies must adopt methods that more accurately reflect true muscle mass, such as the D_3_-creatine dilution method, or further refine existing approaches to better capture functional muscle mass. Importantly, integrating precise assessments of muscle mass with corresponding changes in muscle function will be essential for improving the interpretation of intervention outcomes.

## 6. Clinical Implications

**Figure 5:**
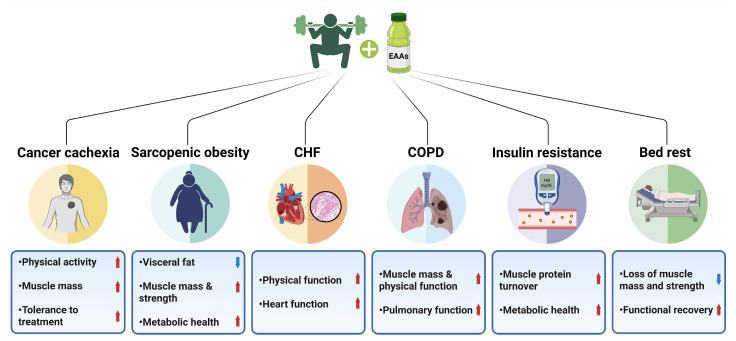
Potential therapeutic impact of balanced essential amino acid supplementation, resistance training or in combination across muscle-wasting conditions. This schematic summarizes the clinical effects of EAA supplementation and RT, administered independently or in combination, across various conditions characterized by muscle wasting and functional decline. EAAs, essential amino acids; RT, resistance training; CHF, chronic heart failure; COPD, chronic obstructive pulmonary disease. Upward and downward arrows (↑, ↓) indicate an increase and a decrease, respectively, in each parameter following EAA supplementation, RT, or their combination. Created in BioRender. Jang, J. (2026) https://BioRender.com/6ut9rhm (accessed on 16 June 2026). Key references supporting the clinical evidence depicted: cancer cachexia [79,80,81]; sarcopenic obesity [82,83,84]; chronic heart failure [85,86,87]; COPD [88,89,90]; insulin resistance [91,92,93]; bed rest [45,70,94,95].

Dietary EAAs alone or in combination with RT can produce clinical benefits beyond improvements in glucose metabolism and insulin sensitivity, primarily through the stimulation of net MPS (MPS minus MPB) in clinical conditions associated with muscle wasting including cancer cachexia, sarcopenia, heart failure, insulin resistance, chronic obstructive pulmonary disease, and bed rest. The potential therapeutic effects of EAAs and RT, administered independently or in combination, are summarized in this section (Figure 5). Before reviewing the evidence, two methodological considerations warrant attention. First, individuals with muscle-wasting conditions frequently fail to meet recommended protein intake levels due to anorexia, dysphagia, and disease-related metabolic dysregulation [75], and inadequate protein intake has been associated with accelerated muscle loss in older adults [76]. EAA supplementation may therefore serve both as an anabolic stimulus and as a means of correcting baseline protein deficiency, with evidence indicating that EAA-based supplements can enhance the anabolic properties of a suboptimal protein intake, particularly when combined with RT [77]. As background dietary protein intake was not consistently reported or controlled across the studies reviewed below, the magnitude of observed EAA supplementation effects should be interpreted with this limitation in mind. Second, the majority of reviewed studies assessed LBM for “muscle mass” by DXA or bioelectrical impedance rather than direct measures of functional muscle mass due to its relatively recent development [64,78]. As LBM is not equivalent to skeletal muscle mass and its accuracy is further limited by hydration status and adiposity, particularly in clinical populations, future studies should consider adopting the D_3_-creatine dilution method or comparable approaches to improve the accuracy of muscle mass assessment and the interpretability of intervention outcomes.

### 6.1. Cancer Cachexia

Cancer cachexia is a multifactorial syndrome characterized by tumor-induced metabolic alterations, systemic inflammation, and reduced nutritional intake, leading to progressive losses of skeletal muscle and fat mass, and ultimately impairing physical function and overall health status [96]. These declines in muscle mass and strength are closely associated with reduced treatment tolerance, increased complications, and higher mortality in cancer patients [97]. Both EAA supplementation and RT demonstrate potential to attenuate muscle deterioration and improve functional outcomes in these populations. EAA supplementation stimulates Wb-PS and supports functional and metabolic outcomes, with evidence showing greater anabolic responses assessed by a stable isotope tracer methodology following ingestion of 14 g of free EAA, compared with an isocaloric non-EAA control beverage in a crossover human study of patients with advanced non-small-cell lung cancer [79], as well as improvements in muscle strength and reductions in oxidative stress following 8 weeks of oral EAA-based amino acid supplementation (8 g/day, twice daily) in an open non-randomized phase II study of cachectic cancer patients across mixed tumor types, with outcomes assessed by dynamometry and blood markers of oxidative stress [80]. Similarly, a feasibility study of progressive RT over 10 weeks (2–3 sessions/week, 50–80% 1RM) in post-treatment lung cancer survivors demonstrated improvements in muscle strength assessed by 1RM and selected physical performance outcomes, with some evidence of increases in LBM assessed by DXA, though the absence of a control group limits interpretation [81]. However, although increases in MPS and muscle strength are consistently observed, evidence supporting long-term maintenance of muscle mass, improved treatment tolerance, or survival remains limited. These studies are characterized by short intervention durations, small and clinically heterogeneous populations, and implementation after the onset of cachexia or in patients with established muscle wasting, which limits the ability to capture the progression of muscle loss and long-term adaptation [79,80,81]. These limitations reflect important gaps in the current evidence base, and caution is warranted in interpreting short-term anabolic responses as indicative of long-term clinical efficacy in this population.

### 6.2. Sarcopenic Obesity

Sarcopenic obesity, defined as the coexistence of excess adiposity and reduced skeletal muscle mass, is associated with impaired physical function and increased cardiometabolic risk, reflecting the combined effects of muscle loss and fat accumulation [98,99]. EAA supplementation stimulates MPS and improves whole-body protein balance in both young and older adults, as demonstrated in an acute human study assessing MPS via the stable isotope tracer methodology following bolus oral EAA (15 g) ingestion without exercise [100]. In obese older adults, a double-blind RCT comparing an EAA-enriched meal replacement against a standard commercial meal replacement over 4 weeks demonstrated improvements in body composition assessed by DXA, including reductions in total and visceral fat, as well as enhanced physical function, as reflected by improved performance on the 6 min walk test [82]. In addition, EAA supplementation (15 g/day, administered as 7.5 g twice daily) for 3 months compared with a control condition increased LBM and basal MPS assessed via a stable isotope methodology in older women [83]. Resistance training is an effective intervention for improving muscle function and body composition in obese older adults. In a 6-month RCT in obese older adults randomized to RT (3 sessions/week, 65–85% 1RM with progressive overload), aerobic exercise, combined exercise, or dietary restriction alone, RT-containing groups demonstrated significant improvements in muscle strength and fat-free mass assessed by DXA, while reductions in fat mass were primarily observed when RT was combined with aerobic exercise [84]. The combined application of RT and leucine-enriched EAA mixture (3 g twice daily) in an RCT of sarcopenic older women demonstrated additive benefits in LBM and functional performance assessed by standardized performance tests compared with either intervention alone [101]. Despite these favorable metabolic and functional adaptations, direct evidence demonstrating consistent increases in skeletal muscle mass in populations specifically defined as sarcopenic obesity remains limited, as most studies focused on MPS, LBM, or functional outcomes rather than direct measures of skeletal muscle mass. Future studies should prioritize well-controlled long-term interventions targeting sarcopenic obesity, with concurrent assessment of muscle mass (not just LBM), adiposity, and functional outcomes to determine whether combined RT and EAA interventions can elicit simultaneous improvements in muscle and fat-related parameters.

### 6.3. Chronic Heart Failure

Chronic heart failure (CHF) is characterized by reduced cardiac output, neurohormonal activation, and systemic inflammation, all of which contribute to metabolic dysregulation and progressive skeletal muscle wasting [102]. These alterations are clinically significant because muscle wasting in CHF is associated with a poor prognosis, including increased mortality risk [103]. EAA supplementation has shown preliminary beneficial effects in specific CHF populations with defined clinical characteristics. In a randomized, double-blind, placebo-controlled trial of elderly CHF outpatients (NYHA class II–III), 30 days of oral amino acid supplementation (4 g twice daily) compared with a placebo improved exercise capacity as assessed by VO2 and the 6 min walk test [85]. In addition, in a randomized controlled trial of stable CHF patients with severe muscle depletion, EAA supplementation (8 g/day) compared with adequate energy–protein intake alone over approximately 4 months improved nutritional and metabolic status as assessed by plasma albumin and arm muscle area, even in the presence of a catabolic condition [86]. In a randomized controlled trial of stable CHF patients (NYHA class II–III), an 8-week isotonic RT program (60% 1RM, 3 sessions/week) compared with usual care improved physical fitness and LV function as assessed by 1RM, a 6 min walk test, and echocardiography [87]. Although EAA supplementation and RT independently improve functional and metabolic outcomes, direct evidence evaluating their combined effects in CHF populations remains limited. Most studies focus on functional and metabolic outcomes, with limited assessment of skeletal muscle mass or protein turnover, and variability in disease severity, treatment, and baseline muscle status may contribute to heterogeneous responses. A systematic review of protein and EAA supplementation in CHF found that all available RCTs had a high risk of bias and that supplementation did not consistently improve muscle strength, though improvements in walking performance, lean body mass, and quality of life were suggested [104]. Future studies should determine whether integrated EAA and RT interventions produce additive benefits on muscle mass and clinically meaningful outcomes, and whether these effects vary according to disease characteristics, with a particular emphasis on longer-duration trials that assess sustained functional and survival outcomes.

### 6.4. Chronic Obstructive Pulmonary Disease

Chronic obstructive pulmonary disease (COPD) is characterized by persistent airflow limitation and systemic manifestations, including chronic inflammation and metabolic dysregulation, which contribute to skeletal muscle loss and impaired physical function [105]. Muscle wasting in COPD is clinically important, as it is associated with reduced functional capacity and increased mortality risk, particularly in patients with moderate to severe disease [105,106]. Balanced EAA supplementation has shown promising beneficial effects in patients with severe COPD. In a randomized placebo-controlled trial of 88 outpatients with GOLD class 3–4 COPD, 12 weeks of oral EAA supplementation (8 g/day) improved daily physical activity assessed by accelerometry, health-related quality of life assessed by the St George’s Respiratory Questionnaire, fat-free mass, muscle strength, arterial oxygen saturation, and serum albumin compared with a placebo [88]. RT has also been shown to improve muscle morphology and function in COPD. A pre–post study of COPD patients, without a randomized control group, demonstrated that 8 weeks of bilateral high-intensity isokinetic knee extensor RT (3 sessions/week, 5 sets of 30 maximal contractions at 180°/s) increased thigh lean mass assessed by DEXA, rectus femoris cross-sectional area, and quadriceps muscle thickness assessed by ultrasound, indicating gains in lower-limb lean mass [89]. In addition, a systematic review and meta-analysis including 18 trials and 750 subjects with advanced COPD found that RT interventions of at least 4 weeks improved dyspnea assessed by the Borg scale, skeletal muscle strength assessed by 1RM or isokinetic dynamometry, and percent-of-predicted FEV_1_ compared with non-exercise controls [90]. Clinical guidelines further support lower-limb RT as a key component of pulmonary rehabilitation because it confers gains in muscle force and contributes to improvements in skeletal muscle adaptations in COPD populations [107]. Nevertheless, most studies have focused on functional and regional outcomes, including muscle strength and thigh muscle size [88,89]. These measures primarily reflect short-term or localized adaptations, and direct evidence linking them to sustained whole-body muscle preservation or long-term clinical outcomes remains limited. In addition, variability in disease severity may contribute to heterogeneous responses. Although both interventions independently demonstrate benefits, evidence for their combined application in COPD remains limited. Future studies should determine whether integrated EAA supplementation and resistance training improve long-term muscle preservation and clinically meaningful outcomes across disease stages.

### 6.5. Insulin Resistance and Type 2 Diabetes Mellitus

Insulin resistance (IR) in skeletal muscle is a central feature of metabolic syndrome and a key contributor to the development of type 2 diabetes mellitus (T2D). Because skeletal muscle is the primary site of glucose disposal from blood, impairments in muscle metabolism contribute directly to poor glycemic control [108]. In addition, insulin regulates protein turnover primarily by suppressing MPB in humans, and this effect is impaired in insulin-resistant states, leading to disruptions in whole-body protein balance [109]. Balanced EAA supplementation has been shown to improve muscle NPB by stimulating MPS and suppressing MPB under acute, controlled metabolic conditions, typically assessed over short-term amino acid infusion or feeding periods in human studies [11]. Preclinical evidence from a mouse model further indicates that EAA ingestion can influence mitochondrial remodeling processes in response to repeated exercise stimuli [5]. RT has been shown to improve insulin-mediated glucose uptake and skeletal muscle metabolic characteristics in patients with T2D. In a controlled intervention study using a within-subject unilateral training design in T2D patients, 6 weeks of one-legged strength training (3 sessions/week) increased insulin-stimulated glucose uptake assessed by a hyperinsulinemic clamp combined with the AV balance, GLUT4 content, and insulin signaling assessed by muscle biopsy, compared with the untrained contralateral leg [91]. In addition, a matched-group RCT comparing 10 weeks of RT (3 sessions/week, 7 upper- and lower-body exercises) versus aerobic exercise in T2D patients demonstrated that RT lowered HbA1c to a greater extent than aerobic training, and a systematic review and meta-analysis further confirmed that structured exercise training is associated with reductions in HbA1c in T2D patients [92,93]. Supporting preclinical evidence from a mouse model of dexamethasone-induced muscle wasting indicates that combined EAA and RT improves muscle function and insulin sensitivity compared with either intervention alone [34]. However, most clinical studies have focused on insulin sensitivity and glycemic control rather than direct measures of muscle mass or long-term outcomes. Although improvements in insulin signaling and HbA1c have been consistently reported [91,92,93], evidence linking these changes to sustained muscle preservation or disease progression remains limited. Evidence for combined EAA and RT in clinical T2D populations is also limited. Future studies should determine whether integrated interventions produce additive benefits on muscle metabolism, glycemic control, and clinically meaningful long-term outcomes.

### 6.6. Muscle and Functional Recovery After Severe Physical Inactivity

Prolonged bed rest due to injury, illness, or surgery leads to significant losses in skeletal muscle mass and function, primarily driven by reductions in MPS and the absence of mechanical loading [70,110]. Physical inactivity also induces anabolic resistance, attenuating the muscle protein synthetic response to protein ingestion during periods of disuse [111]. EAA supplementation has been shown to attenuate disuse-induced muscle loss under controlled conditions. In a randomized placebo-controlled trial, 10 days of bed rest in older adults reduced muscle function and nitrogen balance, whereas EAA supplementation (15 g × 3/day) compared with a placebo improved muscle function assessed by strength testing and the nitrogen balance [45]. In younger individuals, a controlled bed rest study demonstrated that EAA combined with carbohydrate supplementation (16.5 g EAA + 30 g carbohydrate, 3 times daily) compared with no supplementation attenuated LBM loss assessed by a stable isotope tracer methodology and DXA during 28 days of bed rest [70]. In addition, a randomized controlled perioperative trial demonstrated that EAA supplementation (20 g twice daily) following total knee arthroplasty attenuated muscle atrophy assessed by muscle cross-sectional area during the recovery period [112]. RT and mechanical loading interventions play a critical role in preserving muscle tissue volume during periods of inactivity. In a controlled study, 8 weeks of bed rest with or without RT (3 days/week, leg press) demonstrated that the decline in thigh muscle volume was attenuated in the RT condition compared with bed rest alone [94]. Combined interventions further support the complementary roles of EAA and RT. In a randomized study of 28 days of bed rest under energy-deficient conditions, RT (6 days/week, 70–80% 1RM) combined with timed EAA supplementation (15 g/day, administered 5 min before exercise) preserved midthigh muscle area assessed by CT and lower body strength more effectively than EAA alone or RT with post-exercise EAA (3 h after) [95]. However, most evidence is derived from controlled bed rest or perioperative models, which may not reflect the complexity of clinical recovery characterized by ongoing inflammation, variable immobilization, and progressive reloading. In addition, although EAA supplementation improves the nitrogen balance and attenuates the decline in LBM during short-term inactivity [45,70], and RT preserves the muscle volume when applied [94], the early implementation of RT is often limited in clinical settings due to pain, joint instability, and surgical constraints. Furthermore, direct evidence linking these interventions to long-term functional recovery across diverse patient populations remains limited. Future studies should therefore focus on developing phase-specific integrated strategies that optimize the timing of EAA supplementation and the safe initiation of RT to improve muscle mass and long-term functional recovery in postoperative and immobilized populations.

### 6.7. Potential Strategies to Synergize EAA-Based Interventions

Emerging evidence suggests that several physiological and nutritional strategies may enhance the anabolic actions of EAAs. These strategies include enhancing the delivery of EAAs to skeletal muscle, supporting energy for MPS, and reducing inflammation to attenuate anabolic resistance. First, nutrients that improve vascular perfusion may reinforce the anabolic effect of EAAs. It has been shown that pharmacological vasodilation increased muscle blood flow and enhanced insulin-stimulated NPB across the leg, as assessed by the arteriovenous balance in older adults [113], indicating that improved vascular function could potentiate EAA utilization and recovery. In addition to pharmacological agents, nutritional approaches that stimulate nitric oxide (NO) production, such as supplementation with L-arginine or L-citrulline, the (in)direct precursor of NO, may also promote vasodilation and increase skeletal muscle perfusion, thereby supporting EAA transport and anabolic efficacy [114]. However, evidence directly linking improvements in vascular perfusion to enhanced EAA-induced anabolic responses in humans remains unclear. Second, nutrients that support mitochondrial metabolism and cellular energetics, such as L-carnitine and creatine, may further reinforce the effects of EAAs as MPS and MPB are ATP-dependent processes [115,116]. L-carnitine facilitates mitochondrial fatty acid transport and has been associated with enhanced oxidative capacity and reduced muscle damage during recovery [117]. Creatine supplementation improves phosphagen availability and RT adaptations, potentially creating a more favorable energetic environment for EAA-driven MPS and functional improvements [118]. However, the extent to which these energetics-related adaptations enhance EAA-induced anabolic responses remains unclear. Lastly, nutrients that attenuate inflammation may reinforce the anabolic actions of EAAs as increased inflammation in older adults has been suggested to be one of the potential mechanisms that lead to anabolic resistance [119,120]. In this regard, omega-3 fatty acids possess anti-inflammatory and membrane-modulating properties that improve the anabolic sensitivity of muscle tissue [121]. Despite these mechanistic advantages, whether such improvements in anabolic sensitivity translate into a greater anabolic response remains unclear. When provided alongside EAAs, these approaches could amplify anabolic signaling and support long-term muscle maintenance, particularly in aging or catabolic conditions. Overall, despite a strong mechanistic rationale, direct evidence supporting their additive or synergistic effects on EAA-induced anabolic responses in humans remains limited. In particular, whether improvements in vascular perfusion, cellular energetics, or inflammatory status translate into greater NPB or functional recovery when combined with EAAs has not been clearly established.

### 6.8. Practical Considerations for Clinical Application

Although EAA supplementation combined with RT shows considerable promise across a range of clinical conditions, several practical factors must be considered when translating these findings into clinical practice.

Resistance training prescription. While studies directly examining the interaction between specific RT prescription variables and EAA supplementation in humans remain limited, general evidence indicates that moderate-to-high-intensity RT (70–85% 1RM) with progressive overload supports both strength and hypertrophic adaptations [122], and that a weekly set volume is a key determinant of hypertrophy [123]. Eccentric muscle actions may further contribute to hypertrophic adaptation, though evidence remains limited [123]. Future studies should examine how variations in RT prescription interact with EAA supplementation to optimize adaptations across healthy and clinical populations.

EAA dose, composition, and timing. Current evidence suggests that EAA doses typically ranging from 6 to 40 g/day depending on the clinical context are generally effective in stimulating MPS in both healthy and clinical populations. In aging and disease-associated muscle loss, anabolic resistance may attenuate the MPS response to a given EAA dose, necessitating a higher or more targeted EAA intake; older adults require a higher concentration of EAAs compared to younger individuals to elicit an equivalent anabolic response [124]. Notably, however, even low doses of approximately 3.6 g of a leucine-enriched EAA composition have been shown to effectively stimulate MPS in older individuals [125], suggesting that the composition and amino acid profile, rather than dose alone, may be critical determinants of anabolic efficacy. Balanced free-form EAA formulations containing all nine essential amino acids appear more effective than leucine alone. EAA ingestion in close proximity to resistance exercise may further optimize the anabolic response by capitalizing on exercise-induced upregulation of amino acid transporters and mTORC1 signaling [22,23].

Long-term adherence, cost, and palatability. EAA supplementation is generally well tolerated in healthy and clinical populations [126,127]. Adherence to EAA supplementation has been generally high in short-term clinical trials [127], though whether this extends to long-term real-world settings remains to be established, particularly in older adults and those with chronic disease, who may face challenges related to appetite, gastrointestinal comfort, and motivation. While EAA supplements are generally more affordable than pharmacological interventions, cost may still represent a practical barrier to sustained use in some clinical populations. Furthermore, palatability varies across formulations and individuals, and selecting appropriately flavored or unflavored formulations may be important for optimizing long-term adherence in clinical practice.

Safety in specific clinical populations. EAA supplementation is generally safe, with no adverse responses reported in the available literature. However, the safety profile of high-dose or long-term EAA supplementation has not been fully established, and potential adverse effects cannot be excluded, particularly in populations with pre-existing metabolic conditions, such as severe renal or hepatic impairment. In these populations, individualized monitoring and dose adjustment in consultation with a clinical dietitian or physician is advisable.

## 7. Conclusions

Dietary EAAs and RT play an important role in regulating muscle mass via improved muscle protein turnover. In addition, although the underlying mechanisms are yet to be established, EAAs and RT may improve muscle quality through NMJ stability and structural integrity in addition to gains in muscle mass and may also enhance endurance or cardiorespiratory fitness and glucose metabolism through the improvement of mitochondrial quality (via dynamic remodeling). Because EAA supplementation is relatively inexpensive and RT does not require costly medical interventions, EAAs and RT may represent safe, efficient, and cost-effective therapeutic approaches to ameliorate or prevent diseases such as sarcopenia or other muscle wasting diseases. However, the effects of EAAs and RT in various disease states involving loss of muscle mass and physical function are not yet well understood. Future research should focus on elucidating the underlying physiological mechanisms by which EAAs and RT exert their effects in various disease states.

Although promising, the current evidence has several limitations. Most mechanistic insights have been derived from animal and short-term human studies, which restrict the generalizability of these findings to broader clinical populations. In particular, key mechanisms discussed in this review, including NMJ remodeling and PGC-1α-independent mitochondrial biogenesis, remain primarily supported by preclinical evidence, and their relevance to human physiology requires further investigation. Considerable heterogeneity in supplementation protocols, including differences in EAA composition, dosage, and timing, also complicates direct comparisons across studies. Furthermore, most clinical trials included in this review are characterized by small sample sizes, short intervention durations, and reliance on intermediate outcomes such as LBM and muscle strength, rather than long-term functional endpoints or survival outcomes. This limits the ability to draw firm conclusions regarding the sustained clinical efficacy of combined EAA and RT interventions across disease states. Furthermore, long-term data on the efficacy, safety, and sustainability of combined EAA and RT interventions remain limited. Addressing these gaps through well-controlled longitudinal and mechanistic studies will be essential to establish evidence-based recommendations for clinical application.

## Figures and Tables

**Table 1:** Summary of principal mechanistic pathways linking EAA supplementation and resistance training to functional and clinical outcomes.

Pathway/ Mechanism	Primary Stimulus	Molecular Effect	Functional Outcome	Clinical Relevance
mTORC1 [21,22,23]	EAA(leucine), RE	p70S6K1 and 4E-BP1 phosphorylation; upregulation of AA transporters (LAT1, SNAT2, CD98, PAT1)	MPS ↑; muscle hypertrophy	Sarcopenia, cancer cachexia, sarcopenic obesity, CHF, COPD, T2D, bed rest
ERK1/2 [24,25,27]	IGF-1, RE, EAA	rRNA gene expression ↑; eIF4E phosphorylation ↑; translational capacity ↑	MPS ↑ via enhanced translational machinery	Mechanistic basis for anabolic response to RE and EAA; specific clinical translation not yet established
JNK/SMAD [26,28,29]	Mechanical stress	SMAD2 linker phosphorylation; myostatin inhibition; mTORC1 activity ↑	MPB ↓; MPS ↑ (indirect); muscle quality ↑	Mechanistic basis for RE-induced muscle preservation; specific clinical translation not yet established
Mitochondrial biogenesis (PGC-1α-independent) [5,7,48,49]	EAA, endurance exercise	Mitochondrial protein synthesis ↑; biogenesis independent of PGC-1α	Endurance capacity ↑; cardiorespiratory fitness ↑	Primarily preclinical (mouse) evidence; direct human evidence linking EAA supplementation to mitochondrial biogenesis remains limited
Mitochondrial dynamics (mTORC1-DRP1) [5,54,55]	EAA + RT	DRP1 phosphorylation ↑; mitochondrial fission → quality control; mitochondrial protein turnover ↑	Mitochondrial function ↑; endurance capacity ↑	Primarily preclinical (mouse) evidence; whether mTORC1-DRP1 signaling mediates effects in humans requires further investigation
NMJ stability (Agrin/MuSK) [5,6,33,47]	EAA + RT	AChR clustering ↑; MuSK activation; NMJ structural remodeling	Muscle strength and quality ↑ independent of muscle mass	Sarcopenia, muscle quality decline; primarily preclinical (mouse) evidence; human translation not yet established

Abbreviations: EAA, essential amino acid; RE, resistance exercise; RT, resistance training; MPS, muscle protein synthesis; MPB, muscle protein breakdown; mTORC1, mammalian target of rapamycin complex 1; ERK1/2, extracellular signal-regulated kinase 1/2; JNK, c-Jun N-terminal kinase; SMAD, SMAD family member; DRP1, dynamin-related protein 1; NMJ, neuromuscular junction; AChR, acetylcholine receptor; MuSK, muscle-specific kinase; PGC-1α, peroxisome proliferator-activated receptor gamma coactivator 1-alpha; CHF, chronic heart failure; COPD, chronic obstructive pulmonary disease. T2D, type 2 diabetes mellitus. Upward and downward arrows (↑, ↓) indicate an increase and a decrease, respectively. Horizontal arrows (→) indicate that one event leads to the next.

## Data Availability

No new data were created or analyzed in this study. Data sharing is not applicable to this article.
